# Oridonin prevents insulin resistance–mediated cognitive disorder through PTEN/Akt pathway and autophagy in minimal hepatic encephalopathy

**DOI:** 10.1111/jcmm.14546

**Published:** 2019-09-30

**Authors:** Fangfang Wen, Weishan Zhuge, Jian Wang, Xiaoai Lu, Ruimin You, Leping Liu, Qichuan Zhuge, Saidan Ding

**Affiliations:** ^1^ Zhejiang Provincial Key Laboratory of Aging and Neurological Disease Research, Department of Surgery Laboratory the First Affiliated Hospital of Wenzhou Medical University Wenzhou China; ^2^ Key Laboratory of Diagnosis and Treatment of Severe Hepato‐Pancreatic Diseases of Zhejiang Province the First Affiliated Hospital of Wenzhou Medical University Wenzhou China; ^3^ Gastrointestinal Surgery the First Affiliated Hospital of Wenzhou Medical University Wenzhou China; ^4^ Neurosurgery Department the First Affiliated Hospital of Wenzhou Medical University Wenzhou China

**Keywords:** autophagy, cognitive, insulin resistance, minimal hepatic encephalopathy, oridonin, synaptic

## Abstract

Minimal hepatic encephalopathy (MHE) was characterized for cognitive dysfunction. Insulin resistance (IR) has been identified to be correlated with the pathogenesis of MHE. Oridonin (Ori) is an active terpenoid, which has been reported to rescue synaptic loss and restore insulin sensitivity. In this study, we found that intraperitoneal injection of Ori rescued IR, reduced the autophagosome formation and synaptic loss and improved cognitive dysfunction in MHE rats. Moreover, in insulin‐resistant PC12 cells and N2a cells, we found that Ori blocked IR‐induced synaptic deficits via the down‐regulation of PTEN, the phosphorylation of Akt and the inhibition of autophagy. Taken together, these results suggested that Ori displays therapeutic efficacy towards memory deficits via improvement of IR in MHE and represents a novel bioactive therapeutic agent for treating MHE.

## 1 INTRODUCTION

Minimal hepatic encephalopathy (MHE) is a kind of mildest form of hepatic encephalopathy (HE),^1^ which refers to the lack of clinical evidence for hepatic encephalopathy, and the slight alteration in cognitive function will be observed by electrophysiological parameters.^2^ MHE can develop into clinical HE, then more serious changes in co‐ordination and motor activity, slowly declined in intellectual functions and consciousness, which even worse is that progress to coma and death.^3^ If the diagnosis is made ahead of the course of the disease, these clinical features are potentially reversible.^4^ Therefore, the underlying pathogenesis of MHE and the opposite therapeutic drug urgent need solve**.**

It had already proved that fast hyperinsulinaemia has been described in various cirrhotic populations,^5^, ^6^ which is always accompanied by glucose intolerance of varying degrees.^7^, ^8^ IR refers to the coexistence of hyperinsulinaemia and normal or impaired carbohydrate tolerance, and under a pathological condition, the target tissues cannot in response to normal plasma insulin concentration.^9^ Furthermore, study shows that cirrhosis has existed resistance to the glucose‐lowering effect of exogenous insulin.^10^ A great number of studies demonstrated that exposure of hyperinsulinaemia long time will cause peripheral IR and even brain IR.^11^, ^12^, ^13^ Our study had proved that brain IR exists in MHE.^14^ Several studies also demonstrated that brain IR was related to cognitive decline,^15^, ^16^, ^17^ and IR would stimulate onset of synaptic loss.^18^, ^19^ So in this study, the central nervous system (CNS) synaptic deficits can be causally related to MHE‐induced IR.

The study proved that developing IR was closely associated with increased autophagy.^20^ Studies have provided evidence that autophagy is induced in fat, liver, and high‐fat diet‐fed mice,^21^ and autophagy is also induced in the peripheral insulin‐sensitive tissues in response to the IR.^22^ And inhibition of autophagy showed the potential protection of cognitive impairment and spatial learning, which was partly mediated by CREB activation.^23^ The major pathophysiological feature in neurodegenerative diseases is synaptic dysfunction. In vivo, the increased autophagosome formation in the hippocampal CA1 area induced by synaptotoxicity and together with weakening in synaptic plasticity and synapse density.^24^ In addition, activated of Akt by inhibition of phosphatase and tensin homolog (PTEN) has an inhibitory effect on autophagy.^25^ As Akt participates in metabolic signalling pathways for insulin,^26^ PTEN has the function on synaptogenesis.^27^ So we assume that insulin resistant may impair cognitive function of MHE through PTEN/Akt/autophagy pathway.

Terpenoids are promising for treatment of neurodegenerative disorders, especially of cognitive deficits.^28^ Some terpenoids such as ginsenosides, ginkgolides and cannabinoids are exhibiting promising in vitro and in vivo biological activities, which showed the protect way in cognitive.^28^ Oridonin (Ori), an active diterpenoid, is isolated from the traditional Chinese herb Rabdosia rubescens. Studies have found that Ori has various pharmaceutical and biological factions. Ori has already been used in clinical practice. Bohanon et al^29^ reported that Ori inhibited hepatic stellate cell proliferation and fibrogenesis. Recently, the vitro studies found Ori has neuroregulatory effects.^30^, ^31^, ^32^ Also, some studies found the impaired behaviour is significantly restored by Ori treatment.^33^ Moreover, it has demonstrated that Ori can alleviate neurotoxicity and synaptic dysfunction in AD mice,^34^ which means that Ori has the potential application against neurodegenerative disorders. Furthermore, a few studies have investigated that Ori could protect against diabetic nephropathy rats (DN). In addition, terpenoids are able to increase insulin sensitivity in adipose cells^35^ and restore insulin signalling and Akt activities.^35^ Our group had proved that IR took place in MHE.^14^ So we suppose that Ori may improve the MHE by restoring insulin sensitive through PTEN/Akt signalling pathway.

In the present study, we first tested whether Ori had the protective effect on the learning and memory impairment and IR in MHE. Then, we studied the underlying mechanism that Ori could rescue IR‐mediated synaptic loss. Moreover, the study focused on the evaluation of the effects of Ori on MHE via IR‐mediated signalling pathway.

## 2 MATERIALS AND METHODS

### 2.1 Animals

Experimental procedures were carried out in strict accordance with the recommendations in the guide for the Ethics Committees of Wenzhou Medical University. The protocol was approved by the Ethics Committees of Wenzhou Medical University (Protocol Number: SYXK (Zhejiang) 2015‐0009).

Total number of 40 Sprague Dawley rats (experimental animal centre of the Chinese Academy of Sciences in Shanghai) weighing 180‐220 g were used. All experiments were carried out in accordance with the reference set by the Ethics Committees of Wenzhou Medical University regarding the care and use of animals for experimental procedures.

### 2.2 MHE rat models and treatment with Ori

Before the experiments, all rats have two behavioural tests: Y‐maze (YM) and Morris Water Maze (MWM). We obtained the normal values about these behavioural tests. Rats were then randomly divided into two groups: wide type (WT) group (n = 10) and thioacetamide (TAA) group (n = 30). Liver cirrhosis was induced by intraperitoneal injection (ip) of TAA (200 mg/kg in normal saline, Solarbio) twice per week for 11 weeks.

The behavioural manifestations of hepatic encephalopathy in rats that received intraperitoneal injection of TAA evolve four stages: (a) lethargy, (b) mild ataxia, (c) lack of spontaneous movement, loss of righting reflex, but positive response to tail pinch, and (d) coma, and no response to tail pinch. If TAA‐treated rats had one of the above manifestations, it could be diagnosed as overt HE. TAA‐treated rats with no HE symptoms were then again subjected to YM and MWM behavioural tests to confirm whether or not MHE.^36^ MHE rats randomly assigned to two groups (n = 13 in each group), a vehicle control group and an Ori‐treated group, Ori‐treated group were intraperitoneally injected with Ori (5 mg/kg/d) (Shanghai, China) for two weeks, control and MHE groups received saline. At the end of treatment, all groups were subjected to YM and WMW test, respectively.

### 2.3 YM test

Rat was put at the end of an arm and then allowed it to explore the maze freely for 8 minutes, measuring total arm entries and spontaneous alternation percentage (SA %). SA% was defined as a ratio of the arm choices that differed from the previous two choices (‘successful choices’) to total choices during the run (‘total entry minus two’ because the two entries could not be evaluated).^37^

### 2.4 MWM

All groups of rats were trained in a round, water‐filled tub in an environment rich with extra maze cues. An invisible escape platform was located in a fixed spatial location 1 cm below the water surface independent of a subjects start position on a particular trial. Swim latency and path length were determined with a video tracking system (Jiliang, Shanghai, China). Rats were given four trials/day in a 15‐minutes intertrial interval for four consecutive days. For each trial, the maximum trial length was 60 seconds and then calculated the escape latency (EL). On the day 5, the platform was removed and measured the percentage of time spent in the quadrant and the number of platform crossings during task acquisition in 60 seconds. All the data were recorded via visual tracking system.

### 2.5 Detection of fasting glucose and insulin levels

After the behavior tests, each group of rats were fasted for 12 hours, respectively; 3 mL blood sample collected from each rat was centrifuged at 1200 xg for 30 min. The fasting blood glucose levels were determined by a glucose‐oxidase biochemistry analyser and the levels of serum insulin were quantified using specific ELISA kits (Mlbio). All testing processes were carried out according to the manufacturers’ recommendations. Additionally, fasting glucose and insulin were used to calculate HOMA‐IR, an indicator of systemic IR, using the following equation. HOMA‐IR = fasting glucose (mmol/L) × fasting insulin (mU/L)/22.5.

### 2.6 Cells culture and drug treatments

PC12 and N2a cells were purchased from Shanghai Cellular Institute of China Scientific Academy (Shanghai, China), PC12 cells were grown in high glucose DMEM with 10% FBS, and N2a cells were grown in MEM with 10% NEAA and 10% FBS, a mixture of 1% of penicillin/streptomycin in both cells. Cells cultures were incubated at 37℃ in a humid 5% CO_2_/95% air environment.

PC12 and N2a cells were plated at a density of 1 × 10^5^ cells/well on 96‐well plates for 24 hours and serum‐starved for the 12 hours . Then cells were cultured in complete medidum with different high concentration (0.3, 3 and 30 μmol/L) of insulin for 24 hours to induce IR, the response to insulin (100 nmol/L for 10 minutes) was measured by 2‐NBDG uptake. To investigate the effects of Ori on glucose uptake in insulin‐resistant cells, various concentrations of Ori were added to the medium for 24 hours then followed by 100 nmol/L insulin for 10 minutes. The cells were seeded into 6‐well plates at 5 × 10^5^ cells/well for 24 hours and serum‐starved for the next 12 hours. After 12 hours of pre‐treatment with serum‐free DMEM or MEM with high concentration of insulin in the absence or presence of PTEN inhibitor BPV (pic, Selleckchem) 10 μmol/L or Akt inhibitor MK2206 (Selleckchem) 10 μmol/L or autophagy inhibitor 3‐MA (Medchem Express), 0.5 mmol/L was added to the medium 2 hours before Ori (0.5 μmol/L) treatment for 36 hours, and the response to insulin (100 nmol/L for 10 minutes) was measured by 2‐NBDG uptake or RT‐PCR or immunoblotting analysis or immunofluorescence staining.

### 2.7 2‐NBDG uptake

To evaluate the kinetics of 2‐[N‐(7‐nitrobenz‐2‐oxa‐1,3‐diazol‐4‐yl)‐amino]‐2‐deoxy‐D‐glucose (2‐NBDG) (Invitrogen) uptake with increasing cell density, PC12 and N2a cell lines were seeded (1 × 10^5^ cells/well) in clear‐bottomed 96‐well microplates (Costar) in triplicate. After cell treatment, all wells were washed twice with Krebs‐Ringer phosphate buffer (KRP, 1.32 mmol/L NaCl, 4.71 mmol/L KCl2, 47 mmol/L CaCl2, 1.24 mmol/L MgSO4, 2.48 mmol/L Na3PO4, and 10 mmol/L HEPES (pH 7.4)) and incubated with 2‐NBDG (100 μmol/L) for 30 minutes at 37°C in a humidified atmosphere of 5% CO_2_. We stopped the reaction by adding a 2‐fold volume of ice‐cold KRP and the wells were washed again with ice‐cold KRP three times. The fluorescent signal before (autofluorescence) and after adding 100 μmol/L 2‐NBDG was measured using the fluorescence microplate (Thermo) using the excitation wavelength/emission wavelength, Ex/Em 488/520). The net increase in fluorescence was normalized to the lowest signal (0 cells/well), which was taken as the ratiometric quantitation of 2‐NBDG uptake in cells. Finally, the cells in each group were incubated by adding CCK‐8 10 μL for 4 hours which the cells were incubated at a wavelength of 490 nm. CCK‐8 was used to correct the error of fluorescence intensity in each group due to the difference in cell number.

### 2.8 Cell culture and LC3B siRNA‐mediated RNA interference

LC3B‐FAM‐siRNA (sense strand: 5′‐CCCAGUGAUUAUAGAGCGATT‐3′; antisense strand: 5′‐UCGCUCUAUAAUCACUGGGTT‐3′), a target‐specific 21‐nucleotide siRNA designed to silence gene expression, and FAM labelled negative control siRNA (sense strand: 5′‐UUCUCCGAACGUGUCACGUTT‐3′; antisense strand: 5′‐ACGUGACACGUUCGGAGAATT‐3′) were purchased from GenePharma. We also used two other siRNA sequences, sense strand: 5′‐GGAGCUUUGAACAAAGAGUTT‐3′; antisense strand: 5′‐ACUCUUUGUUCAAAGCUCCTT‐3′ and sense strand: 3′‐GCAGCUCAAUGCUAACCAATT‐5′; antisense strand: 3′‐UUGGUUAGCAUUGAGCUGCTT‐5′, for silencing the LC3B gene. SiRNA transfection experiments were performed according to the manufacturer's instructions. Briefly, PC12 cells were seeded at a density of 1 × 10^5^ cells per well in 6‐well plates. Twelve hours after seeding, cells were transfected with control and LC3B siRNA duplexes (20 nmol/L) using Lipofectamine^®^ 2000. Following 4‐6 hours of incubation, and then were observed under a fluorescence microscope (Leica Microsystems) to identify the transfection efficiency. PC12 cells were transfected with control siRNA or 20 nmol/L LC3B siRNA construct and treated with insulin (3 μmol/L) for 24 hours. Finally, culture mediums are in the absence or presence of Ori (0.5 μmol/L) for another 24 hours.

### 2.9 Immunoblotting (IB)

Tissue homogenates of the hippocampus and cerebral cortex of rats, PC12 and N2a cells were harvested in a lysis buffer (Beyotime Biotechnology). In brief, the total amount of protein was determined by bicinchoninic acid (BCA) protein assay (Beyotime Biotechnology). Samples (40 μg protein) were electroblotted to PVDF membrane, which were blocked by incubation in 5% non‐fat dry milk dissolved in TBS‐T (150 mmol/L NaCl, 50 mmol/L Tris, 0.05% Tween‐20). Following transfer, proteins were probed using a primary antibody: synapsin I, PSD95, pAkt (Ser473), Akt, PTEN, Beclin1, LC3B, GAPDH (Abcam). Then, horseradish peroxidase‐conjugated anti‐rabbit secondary antibody was used.

### 2.10 RT‐PCR

The mRNA was extracted from the PC12 and N2a cells using RNA simple Total RNA Kit (Thermo). cDNA synthesis by reverse transcription and quantitative real‐time PCR were performed as protocol. The expression of Beclin1 and LC3B genes were determined by real‐time PCR using SYBR Premix Ex Taq (Roche Diagnostics) and 7500 fast PCR machine (Applied Biosystems). Target gene expression levels were calculated after normalization to the standard housekeeping gene GAPDH, using the 2^−ΔΔCT^ method as protocol. The PCR primer (Sangon Biotech) sets are listed below. Beclin1 (mouse), forward: 5′‐GGACCAGGAGGAAGCTCAGTACC‐3′; reverse: 5′‐CGCTGTGCCAGATGTGGAAGG‐3′. Beclin1 (rattus norvegicus), forward: 5′‐AGGAGTTGCCGTTGTACTGTTCTG‐3′; reverse: 5′‐TGCCTCCAGTGTCTTCAATCTTGC‐3′. LC3B (mouse), forward: 5′‐AGGCCAGGTCTCCGCTTGTC‐3′, reverse: 5′‐GGAGCAGGCATGGACCAGACAG‐3′. LC3B (rattus norvegicus), forward: 5′‐TGAAGGCAGCAACAGGAAGAG‐3′, reverse: 5′‐TGGCTTTCCGTGACAGAG‐3′. PSD95, forward: 5′‐TCCAGTCTGTGCGAGAGGTAGC‐3′, reverse: GGACGGATGAAGATGGCGATGG. synapsin I, forward: 5′‐GTCCTCATTCGTGCTGCCTGTG‐3′, 5′‐AGGAGTGGAGGTTGGAGGAAGATG‐3′. GAPDH (mouse), forward: 5′‐AGGCCGGTGCTGAGTATGTC‐3′; reverse: 5′‐TGCCTGCTTCACCACCTTCT‐3′. GAPDH (rattus norvegicus), forward: 5′‐TTCCTACCCCCAATGTATCCG‐3′; reverse: 5′‐CATGAGGTCCACCACCCTGTT‐3′.

### 2.11 Immunofluorescence staining

For brain tissues: four‐micron frozen cortical sections fixed in acetone or 4% formaldehyde. For cells cultured on glass coverslips were fixed with 4% paraformaldehyde for 30 minutes and then treated with 0.1% Triton X‐100 for 20 minutes at room temperature. Blocking was achieved with PBS containing 5% normal goat serum for 1 hour at room temperature. Sections were then incubated overnight at 4°C with the following primary antibodies: total‐Akt and pAkt‐Ser473 (Abcam), PSD95 (Abcam), synapsin I (Abcam), microtubule‐associated protein 2 (Map2) (Abcam), Beclin1 (Abcam); LC3B (Abcam). Binding of primary antibodies was detected by incubating the sections for 30 minutes with Alexa Fluor 488 (green)/Alexa Fluor 594 (red) conjugated secondary antibody (Abcam), or with horseradish peroxidase (HRP) ‐labelled secondary antibody (Abcam) and were visualized by diaminobenzidine (DAB).

### 2.12 Electron microscopy

All rats were exposed to deep anaesthesia and then were perfused transcardially with 2% glutaraldehyde and 3% paraformaldehyde in PBS. Hippocampal slices were post‐fixed in cold 1% OsO_4_ for 1 day. Samples were prepared and examined using standard procedures. Ultrathin sections (90 nm) were stained with uranyl acetate and lead acetate. Finally, samples were viewed at 100 kV in a JEOL 200CX electron microscope. Synapses were identified by the presence of synaptic vesicles and post‐synaptic densities, and autophagosomes.

### 2.13 Golgi stain

Rats’ brains were fixed in 10% formalin for 24 hours and then immersed in 3% potassium bichromate for a week in the dark. The solution was changed every 3 days. Then, the brains were incubated in 2% silver nitrate solution for 24 hours in the dark. Vibratome sections were cut at 50‐80 mm; air‐dried for 20 minutes, dehydrated through 75, 95 and 100% ethanol, and cleared in xylene, then coverslipped. For measurement of spine density, only spines that emerged perpendicular to the dendritic shaft were counted.

### 2.14 Statistical analysis

Data are presented as mean ± SD. The statistical significance between group comparisons was determined by one‐way analysis of variance (ANOVA). Values of *P* < .05 or *P* < .01 were considered to be statistically significant.

## 3 RESULTS

### 3.1 Ori rescues memory deficits in MHE rats

**Figure 1:**
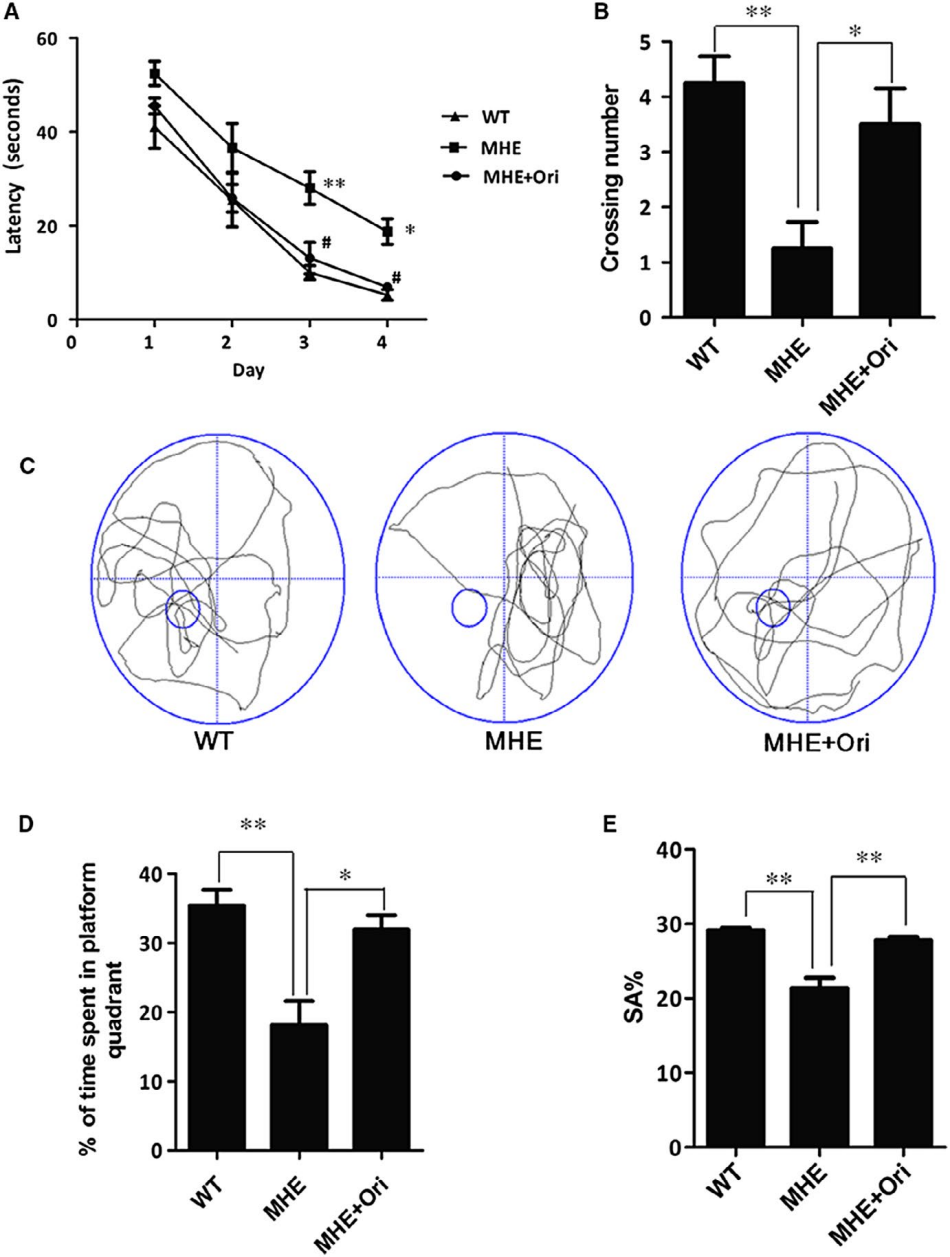
Ori Rescues Memory Deficits in MHE rats. A, EL of MWM in MHE rats treated with Ori.*P < .05, **P < .01 vs WT rats, #*P* < .05 vs MHE rats. B, The number of platform crosses of MWM in MHE rats treated with Ori. C, Representative swim paths during the spatial probe test of MWM in MHE rats treated with Ori. D, Percentage of time in the target quadrant during the probe trial test of MWM in MHE rats treated with Ori. E, SA% in YM of MHE rats treated with Ori. Data are shown as mean ± SD (n = 6). *P < .05, **P < .01

Studies found the impaired behaviour is significantly restored by Ori treatment.^33^ Therefore, we investigated whether Ori had the effect on the cognitive function in MHE rats. Hippocampal‐dependent spatial memories of groups were tested using the MWM. ANOVA (Group Training Day) on EL revealed a main effect of training day. MHE rats exhibited latent memory deficits: a significant delay in EL on the third and fourth day places navigation training compared to the WT rats (*P* < .01, *P* < .05, Figure 1 A), while MHE rats after Ori (5 mg/kg) administration notably displayed the decrease in the EL on the third and fourth day (*P* < .05, Figure 1 A). All groups were subjected to the test in which the target platform was removed on the next day after the end of navigation training, in order to assess the spatial memory more directly. The number of platform crossings and time spent in the target quadrant were significantly increased in MHE rats compared to WT rats (*P* < .01, *P* < .01, Figure 1 B,C,D). Moreover, Ori‐treated rats spent more crossing number and more time in the target quadrant than the MHE rats (*P* < .05, *P* < .05, Figure 1 B,C and D).

Next, the working memory was tested using YM. The results showed that MHE rats had lower percentage of SA % than those of WT rats (*P* < .05, Figure 1 E). However, Ori administration significantly increased the SA% (*P* < .05) (*P* < .05, Figure 1 E). These results indicated that the treatment of Ori could restore the cognitive impairment of MHE rats.

### 3.2 Ori improves IR in MHE rats

**Figure 2:**
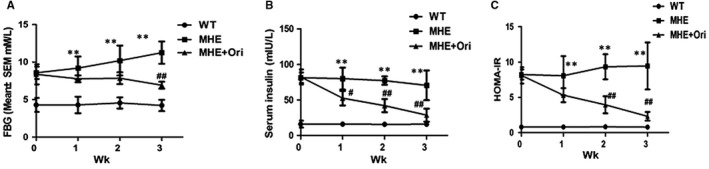
Ori improves IR in MHE rats. A, Assay for fasting blood glucose levels by using glucose‐oxidase biochemistry analyser in MHE rats treated with Ori for 3 wk. B, Assay for fasting serum insulin levels by using ELISA kits in MHE rats treated with Ori for 3 wk. C, Assay for HOMA‐IR was calculated from fasting glucose and insulin levels in MHE rats treated with Ori for 3 wk. Data are shown as mean ± SEM (n = 6). **P < .01 vs WT rats; #*P* < .05, ##*P* < .01 vs MHE rats

It had proved that IR occurred in MHE rats and induced cognitive impairment^14^ and Ori could reduce blood glucose levels and alleviated IR.^38^, ^39^, ^40^ As seen from Figure 2 A,B and C, fasting glucose levels and serum insulin were significantly increased (*P* < .01, *P* < .01), and insulin sensitivity as evaluated by the HOMA‐IR index was markedly deteriorated in MHE rats (*P* < .01), indicating IR in MHE rats. Then, we tested the effects of Ori on fasting glucose levels, serum insulin and HOMA‐IR of MHE rats from first to third week. Ori reduced fasting glucose levels notably only after three weeks later (*P* < .01, Figure 2 A). And Ori‐treated MHE rats reduced serum insulin levels with time‐dependent manner (*P* < .05 vs 1st week, *P* < .01 vs 2nd and 3rd week, Figure 2 B). HOMA‐IR index of Ori‐treated MHE rats was markedly reduced after two and three weeks later compared to MHE rats (*P* < .01, Figure 2 C). These results indicated the effect of Ori on insulin sensitivity in vivo.

### 3.3 Ori promotes glucose uptake in insulin‐resistant PC12 and N2a cells

**Figure 3:**
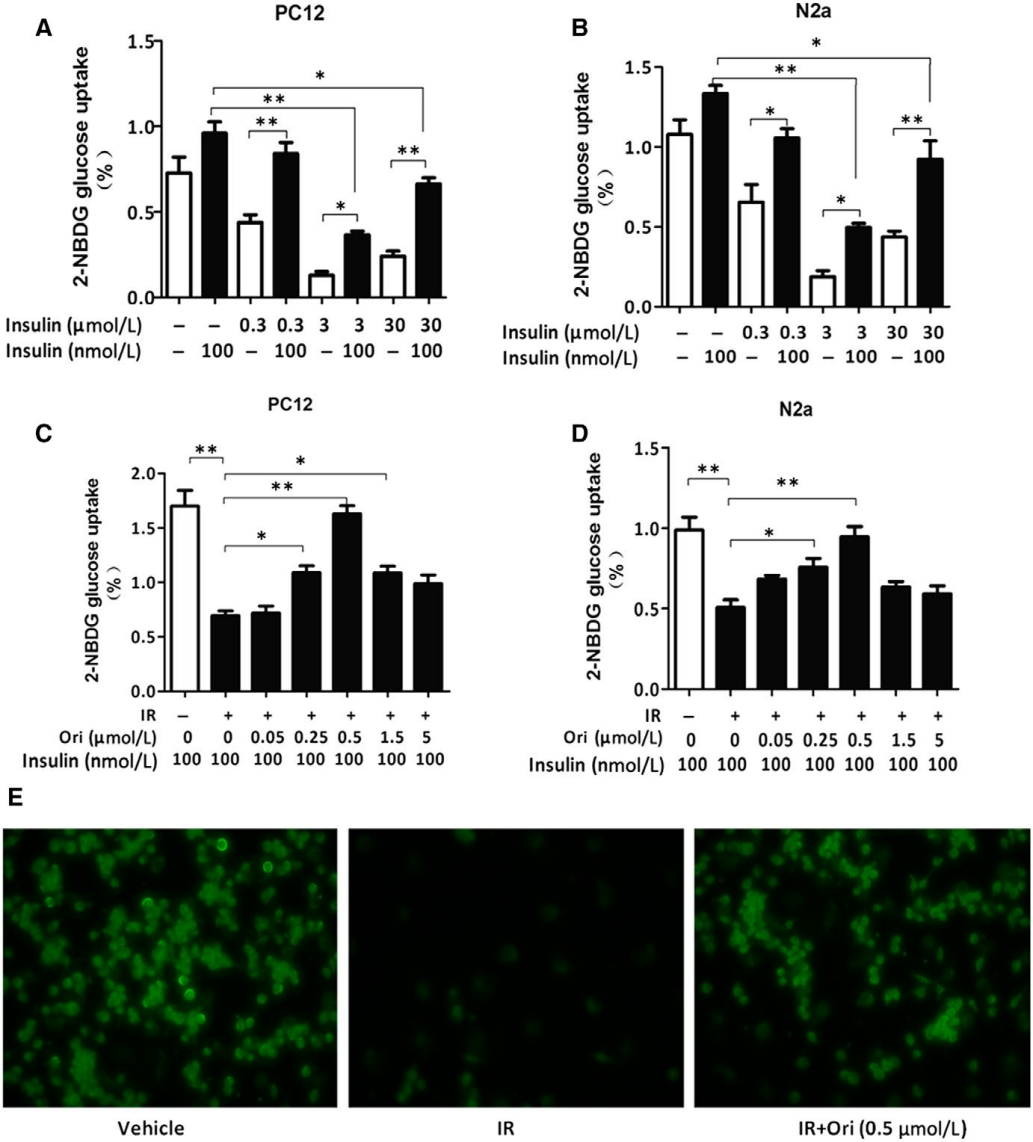
Ori promotes glucose uptake in insulin‐resistant cells. A‐B, 2‐NBDG uptake assay of PC12 and N2a cells stimulated with 100 nmol/L insulin in pre‐incubation of 0.3, 3 or 30 µmol/L insulin by using a fluorometric plate reader. C‐D, 2‐NBDG uptake assay of PC12 and N2a cells treated with various concentration of Ori (0.05, 0.25, 0.5, 1.5 or 5 µmol/L) in the pre‐incubation of 3 µmol/L insulin together with 100 nmol/L insulin by using a fluorometric plate reader. E, Uptake of fluorescent 2‐NBDG assay for PC12 cells treated with 0.5 µmol/L Ori in the pre‐incubation of 3 µmol/L insulin together with 100 nmol/L insulin by using inversed fluorescent microscope. Data are shown as mean ± SD. *P < .05, **P < .01. Scale bar, 25 μmol/L

Two insulin‐resistant neuron models (insulin‐resistant PC12 cells and N2a cells) were established to examine the effect of Ori on IR in vitro using the 2‐NBDG uptake assay. First, we investigated optimal concentration of insulin for establishment of the insulin‐resistant cells models. In PC12 cells, under normal condition, 100 nmol/L insulin stimulated a significant increase of 2‐NBDG uptake compared to the basal cellular level (*P* < .05, Figure 3 A). Under 0.3 μmol/L concentration of insulin condition, 100 nmol/L insulin stimulated an elevation of 2‐NBDG uptake compared to the basal cellular level (*P* < .01, Figure 3 A) and 100 nmol/L insulin caused no significant effect on 2‐NBDG uptake compared to normal condition (Figure 3 A). Under 3 μmol/L concentration of insulin condition, insulin caused a slight but notable increase in 2‐NBDG uptake compared to the basal cellular level (*P* < .05, Figure 3 A), and results showed that insulin failed to enhance 2‐NBDG uptake compared to normal condition (*P* < .01, Figure 3 A). Under 30 μmol/L concentration of insulin condition, insulin caused a significant increase in 2‐NBDG uptake compared to the basal cellular level (*P* < .01, Figure 3 A); however, insulin was incapable to increase 2‐NBDG uptake compared to normal condition (*P* < .05, Figure 3 A). In N2a cells, under normal condition, 100 nmol/L insulin stimulated a notable increase of 2‐NBDG uptake compared to the basal cellular level (*P* < .05, Figure 3 B). Under 0.3 μmol/L concentration of insulin condition, insulin stimulated an increase of 2‐NBDG uptake compared to the basal cellular level (*P* < .05, Figure 3 B), and insulin stimulated no significant effect in 2‐NBDG uptake compared to normal condition (Figure 3 B). Under 3 μmol/L concentration of insulin condition, insulin caused a slight but significant increase in 2‐NBDG uptake compared to the basal cellular level (*P* < .05, Figure 3 B), and results showed that insulin was incapable to enhance 2‐NBDG uptake compared to normal condition (*P* < .01, Figure 3 B). Under 30 μmol/L concentration of insulin condition, insulin caused an obvious increase in 2‐NBDG uptake compared to the basal cellular level (*P* < .01, Figure 3 B), however, insulin was unable to increase 2‐NBDG uptake compared to normal condition (*P* < .05, Figure 3 B). So it was suggested that 3 μmol/L high insulin could induce the establishment of the insulin‐resistant neuron model.

Then, after incubation with 3 μmol/L insulin for 24 hours, PC12 and N2a cells were treated with different concentrations of Ori (0.05, 0.25, 0.5, 1.5, or 5 μmol/L). For insulin‐resistant PC12 cells, insulin was unable to elevate glucose uptake compared to normal condition (*P* < .01, Figure 3 C). We found that the treatment of Ori significantly increased insulin‐stimulated 2‐NBDG uptake with dose‐dependent manner. 0.05 μmol/L Ori had no significant effect on insulin‐stimulated 2‐NBDG uptake, at 0.25 μmol/L Ori notably increased insulin‐stimulated 2‐NBDG uptake, and 0.5 μmol/L Ori showed greater and maximal increase in insulin‐stimulated 2‐NBDG uptake, while Ori at concentration of 1.5 and 5 μmol/L did not change insulin‐stimulated 2‐NBDG uptake (*P* < .05, *P* < .01 Figure 3 C). For N2a cells, under IR condition, insulin was unable to significantly stimulate glucose uptake compared to normal condition (*P* < .01, Figure 3 D), and addition of 0.05 μmol/L Ori did not significantly increase insulin‐stimulated 2‐NBDG uptake, 0.25 μmol/L Ori markedly increased insulin‐stimulated 2‐NBDG uptake, and 0.5 μmol/L Ori caused greater and maximal increase in insulin‐stimulated 2‐NBDG uptake, while 1.5 and 5 μmol/L Ori also had no effect on insulin‐stimulated 2‐NBDG uptake (*P* < .05, *P* < .01, Figure 3 D). So it is suggested that 0.5 μmol/L Ori exerts the optimal effect in insulin‐resistant neuron model.

### 3.4 Ori attenuates autophagy in insulin‐resistant cells

**Figure 4:**
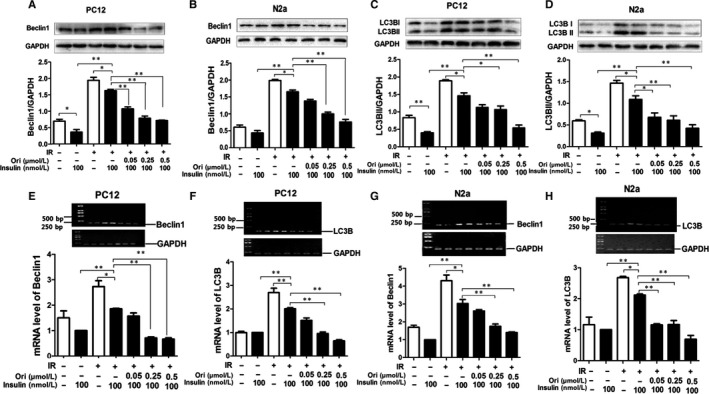
Ori attenuates autophagy in insulin‐resistant cells. A,B, IB analysis of lysates of PC12 (A) and N2a (B) cells treated with various concentration of Ori (0.05, 0.25, 0.5, 1.5 or 5 µmol/L) in the pre‐incubation of 3 µmol/L insulin together with 100 nmol/L insulin using anti‐Beclin1/GAPDH antibodies and subsequent densitometry. C‐D, IB analysis of lysates of PC12 (C) and N2a (D) cells treated with various concentration of Ori (0.05, 0.25, 0.5, 1.5 or 5 µmol/L) in the pre‐incubation of 3 µmol/L insulin together with 100 nmol/L insulin using anti‐LC3B/GAPDH antibodies and subsequent densitometry. E‐H, RT‐PCR analysis for Beclin1/LC3B mRNA of PC12 (E, F) and N2a (G, H) cells treated with various concentration of Ori (0.05, 0.25, 0.5, 1.5 or 5 µmol/L) in the pre‐incubation of 3 µmol/L insulin together with 100 nmol/L insulin. Data are shown as mean ± SD. *P < .05, **P < .01

Additionally, the onset of IR could lead to the induction of autophagy.^41^ Thus, we examined whether Ori had the effect on the IR‐induced autophagy in neurons. For PC12 cells, under normal condition, insulin stimulated a significant decrease in Beclin1 compared to the basal cellular level By IB analysis (*P* < .05, Figure 4 A). Under high concentration of insulin condition, 100 nmol/L insulin caused slight but significant decrement of Beclin1 expression compared to basal cellular level (*P* < .05, Figure 4 A); however, insulin was incapable to decrease Beclin1 expression compared to normal condition (*P* < .05, Figure 4 A), and treatment of Ori at 0.05, 0.25 and 0.5 μmol/L, insulin caused a significant decrease in Beclin1 (*P* < .01, Figure 4 A). For N2a cells, under normal condition, insulin stimulated a minor but no significant reduction of Beclin1 compared to the basal cellular level (*P* > .05, Figure 4 B). Under high concentration of insulin condition, insulin caused a slight decrease in Beclin1 expression compared to the basal cellular level (*P* < .05, Figure 4 B), and insulin failed to decrease Beclin1 expression compared to normal condition (*P* < .01, Figure 4 B), and treated Ori at concentration of 0.25 and 0.5 μmol/L, insulin caused a marked attenuation in Beclin1 expression (*P* < .01, Figure 4 B).

Then, we assayed LC3B expression, under normal condition, insulin stimulated a decrease under the basal cellular level in LC3B (*P* < .01, Figure 4 C). Under high concentration of insulin condition, insulin did not induce notable attenuation of LC3B expression compared to the basal cellular level, while insulin was unable to decrease LC3B expression compared to normal condition (*P* < .01, Figure 4 C), and treatment of Ori at 0.25 and 0.5 μmol/L caused notable decrease in LC3B with insulin stimulation in PC12 cells (*P* < .05, *P* < .01, Figure 4 C). For N2a cells, under normal condition, insulin caused a significant decrease in LC3B level compared to the basal cellular level (*P* < .05, Figure 4 D). Under high concentration of insulin condition, insulin caused a tiny but significant decrement of LC3B expression compared to basal level (*P* < .05, Figure 4 D), however, insulin was unable to decrease LC3B expression compared to normal condition (*P* < .01, Figure 4 D), and addition of Ori at concentration of 0.05, 0.25 and 0.5 μmol/L, significantly attenuated LC3B expression (*P* < .05, *P* < .01, Figure 4 D).

Then, we investigated the mRNA expressions of the Beclin1 and LC3B. For PC12 cells, under normal condition, insulin stimulated a significant decrease in Beclin1 mRNA as compared with the basal cellular level (*P* < .05, Figure 4 E). Under high insulin condition, insulin caused a minor but marked decrement of Beclin1 mRNA compared to basal level (*P* < .05, Figure 4 E), and insulin failed to reduce Beclin1 mRNA compared to normal condition (*P* < .01, Figure 4 E), and treatment of Ori at 0.25 and 0.5 μmol/L markedly decreased in Beclin1 mRNA under high concentration of insulin condition (*P* < .01, Figure 4 E). Similarly, under normal condition, insulin stimulated a significant reduction in LC3B mRNA compared to the basal cellular level (*P* < .05, Figure 4 F). Under high concentration of insulin condition, insulin caused a decrease in LC3B mRNA compared to basal level (*P* < .05, Figure 4 F), insulin failed to decrease LC3B mRNA compared to normal condition (*P* < .01, Figure 4 F), and treatment of Ori at 0.25 and 0.5 μmol/L, insulin caused significant decreases in LC3B mRNA (*P* < .01, Figure 4 F). For N2a cells, under normal condition, insulin stimulated a notable decrease in Beclin1 mRNA compared to the basal cellular level (*P* < .05, Figure 4 G). Under high concentration of insulin condition, insulin caused a tiny but obvious decrement of Beclin1 mRNA compared to basal level (*P* < .05, Figure 4 G), while insulin was unable to decrease Beclin1 mRNA compared to normal condition (*P* < .01, Figure 4 G), and Beclin1 mRNA was notably decreased by addition of Ori at concentration of 0.25 and 0.5 μmol/L (*P* < .01, Figure 4 G). Then, insulin stimulated an obvious attenuation in LC3B mRNA expression under normal condition compared to the basal cellular level (*P* < .05, Figure 4 H). Under high concentration of insulin condition, insulin stimulated a slight but no obvious decrease in LC3B mRNA level compared to basal level (Figure 4 H), insulin failed to decrease LC3B mRNA compared to normal condition (*P* < .01, Figure 4 H), and exposure with Ori at concentration of 0.05, 0.25 and 0.5 μmol/L caused notable decreases in LC3B mRNA level (*P* < .01, Figure 4 H).

### 3.5 Ori activates PTEN/AKT pathway to reduce autophagy in insulin‐resistant cells

**Figure 5:**
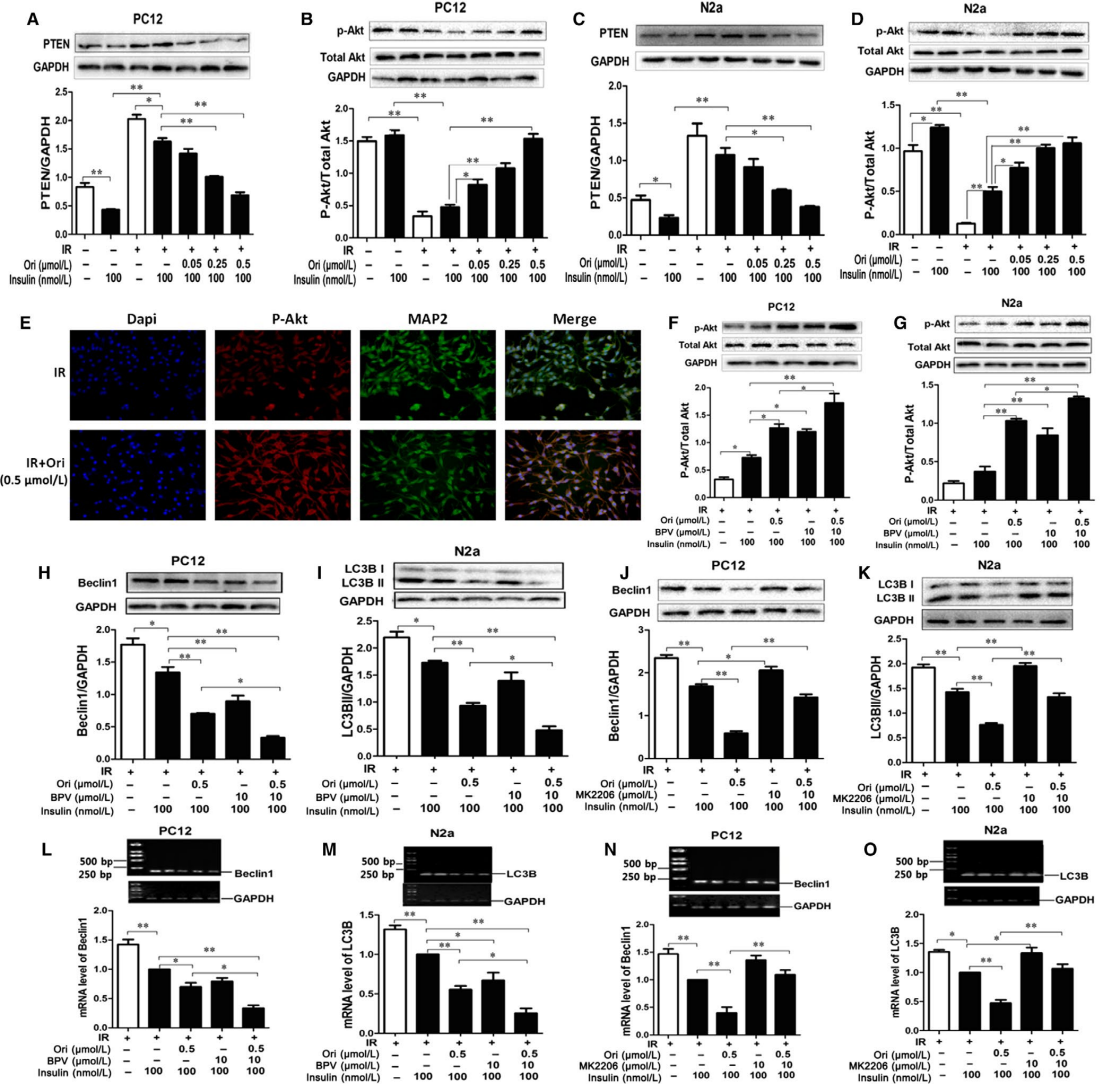
Ori activates PTEN/AKT pathway to reduce autophagy in insulin‐resistant cells. A‐D, IB analysis of lysates of PC12 (A, B) and N2a (C, D) cells treated with various concentration of Ori (0.05, 0.25, 0.5, 1.5 or 5 µmol/L) in the pre‐incubation of 3 µmol/L insulin together with 100 nmol/L insulin using anti‐PTEN/GAPDH and anti‐pAkt(Ser473)/total‐Akt antibodies and subsequent densitometry. E, Double immunofluorescence staining of PC12 cells treated with 0.5 µmol/L Ori in the pre‐incubation of 3 µmol/L insulin together with 100 nmol/L insulin using antibodies against pAkt (red), MAP2 (green). F‐K, IB analysis of lysates of PC12 (F, H, J) and N2a (G, I, K) cells treated with 0.5 µmol/L Ori in the pre‐incubation of 3 µmol/L insulin together with 100 nmol/L insulin in the presence or absence of BPV or MK2206 using anti‐pAkt (Ser473)/total‐Akt (F, G), anti‐Beclin1/GAPDH (H, J), anti‐LC3B/GAPDH (I, K) antibodies and subsequent densitometry. L‐O, RT‐PCR analysis for Beclin1 (L, N)/LC3B (M, O) mRNA of PC12 (L, N) and N2a (M, O) cells treated with 0.5 µmol/L Ori in the pre‐incubation of 3 µmol/L insulin together with 100 nmol/L insulin in the presence or absence of BPV or MK2206. Data are shown as mean ± SD. *P < .05, **P < .01. Scale bar, 25 μm. MRGD, merged image

Additionally PTEN acts as an inhibitor of Akt signalling 42 and PTEN was widely implicated as a negative regulator of Akt signalling.^43^ Furthermore, PTEN negatively affected insulin sensitivity,^44^, ^45^ and Akt participated in metabolic signalling pathways for insulin.^26^ So we investigated the effect of Ori on the IR‐mediated activity of PTEN/Akt signalling. By IB analysis, in PC12 cells, insulin stimulated an obvious reduction in PTEN expression under normal condition (*P* < .01, Figure 5 A). Under high concentration of insulin condition, 100 nmol/L insulin caused a minor but significant decrement of PTEN expression compared to basal level (*P* < .05, Figure 5 A), insulin failed to reduce PTEN expression compared to normal condition (*P* < .05, Figure 5 A), and treatment of Ori at 0.25 and 0.5 μmol/L caused marked decreases in PTEN expression (*P* < .01, Figure 5 A). Next, phosphorylation of Akt was examined, and under normal condition, insulin stimulated a light increase over the basal cellular level in the phosphorylation of Akt (*P* > .05, Figure 5 B). However, under high concentration of insulin condition, 100 nmol/L insulin did not stimulate p‐Akt expression compared to basal level, insulin was also unable to phosphorylate Akt compared to normal condition (*P* < .01, Figure 5 B), and treatment of Ori at concentrations of 0.05, 0.25 and 0.5 μmol/L, significantly phosphorylated Akt (*P* < .05, *P* < .01, Figure 5 B). For N2a cells, under normal condition, insulin stimulated a notable reduction in PTEN expression under the basal cellular level (*P* < .05, Figure 5 C). Under high concentration of insulin condition, insulin caused a slight attenuation of PTEN expression compared to basal level (*P* > .05, Figure 5 C), insulin was unable to decrease PTEN expression compared to normal condition (*P* < .01, Figure 5 C), and the expression of PTEN was significantly decreased by addition of Ori at concentration of 0.25 and 0.5 μmol/L (*P* < .05, *P* < .01, Figure 5 C). Then, insulin stimulated an obvious increase over the basal cellular level in the phosphorylation of Akt under normal condition (*P* < .05, Figure 5 D). However, under high concentration of insulin condition, insulin stimulated a tiny but notable expression of p‐Akt compared to basal level (*P* < .05, Figure 5 D), insulin failed to phosphorylate Akt compared to normal condition (*P* < .01, Figure 5 D), and addition of Ori at concentrations of 0.05, 0.25 and 0.5 μmol/L caused an obvious phosphorylation of Akt (*P* < .05, *P* < .01, Figure 5 D). IF staining showed the p‐Akt immunofluorescences were markedly increased by 0.5 μmol/L Ori treatment in insulin‐resistant PC12 cells (Figure 5 E).

Then, we verified the effect of Ori on Akt activity via PTEN, treatment of BPV further enhanced Ori‐induced Akt phosphorylation in insulin‐resistant PC12 cells (*P* < .05, Figure 5 F) and in insulin‐resistant N2a cells (*P* < .05, Figure 5 G) by IB analysis. These results indicated Ori could rescue the PTEN/Akt pathway in insulin‐resistant cells.

As PTEN has been widely recognized as a positive regulator of autophagy through inhibition of Akt,^46^ we addressed whether Ori elicited IR‐mediated autophagy via PTEN/Akt signalling pathway. Insulin‐resistant PC12 and N2a cells were pre‐treated with 10 μmol/L BPV or 10 μmol/L MK2206 for 2 hours followed by the addition of 0.5 μmol/L Ori for 36 hours. As expected, treatment of BPV significantly enhanced the Ori‐induced decrease in Beclin1 expressions in insulin‐resistant PC12 cells (*P* < .05, Figure 5 H), and BPV also notably amplified attenuation of LC3B expression in insulin‐resistant N2a cells exposed to Ori by IB analysis (*P* < .05, Figure 5 I). However, the treatment of MK2206 markedly abated the Ori‐induced decrease in the expression of Beclin1 in insulin‐resistant PC12 cells (*P* < .01, Figure 5 J), and MK2206 significantly inhibited the reduction of the expression of LC3B in Ori‐treated insulin‐resistant N2a cells (*P* < .01, Figure 5 K) by IB analysis. RT‐PCR analysis showed that treatment of BPV caused further reduction of Beclin1 mRNA in Ori‐treated insulin‐resistant PC12 cells (*P* < .01, Figure 5 L), and the attenuation of LC3B mRNA was markedly enhanced by BPV in insulin‐resistant N2a cells exposed to Ori (*P* < .05, Figure 5 M). However, the treatment of MK2206 notably abated the Ori‐induced decrease in Beclin1 mRNA in insulin‐resistant PC12 cells (*P* < .01, Figure 5 N), and in insulin‐resistant N2a cells, MK2206 obviously blocked the Ori‐induced decrease in LC3B mRNA (*P* < .01, Figure 5 O). They suggested that Ori could improve IR‐mediated PTEN/p‐Akt/autophagy signalling.

**Figure 6:**
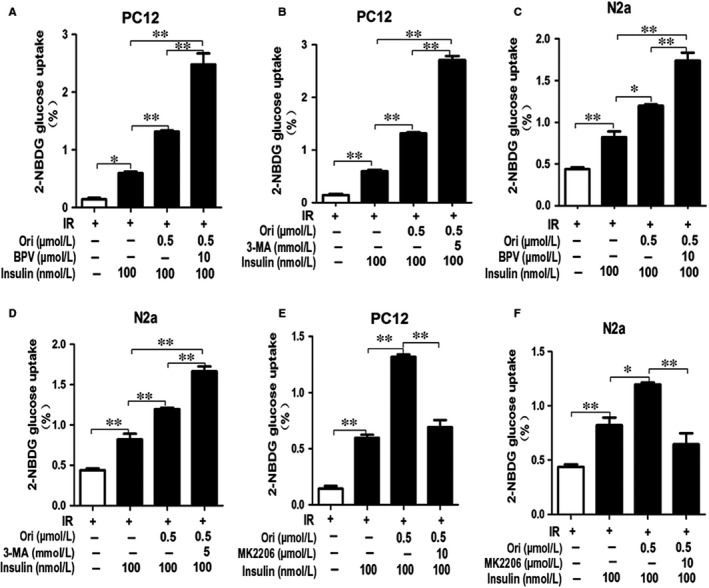
Ori stimulated 2‐NBDG uptake through PTEN/Akt/autophagy pathway in insulin‐resistant cells. A‐F, 2‐NBDG uptake assay of PC12 (A, B, E) and N2a (C, D, F) cells treated with 0.5 µmol/L Ori in the pre‐incubation of 3 µmol/L insulin together with 100 nmol/L insulin in the presence or absence of BPV or 3‐MA or MK2206 by using fluorometric plate reader. Data are shown as mean ± SD. *P < .05, **P < .01

Furthermore, we confirmed whether the effect of Ori on glucose uptake was associated with PTEN/Akt/autophagy signalling pathway. Insulin‐resistant PC12 and N2a cells were pre‐treated with BPV, MK2206 or 3‐MA for 2 hours followed by the addition of 0.5 μmol/L Ori for 36 hours. As expected, pre‐treatment of BPV or 3‐MA caused further elevation of 2‐NBDG uptake in Ori‐treated insulin‐resistant PC12 cells (*P* < .01, Figure 6 A,B), and in Ori‐treated insulin‐resistant N2a cells (*P* < .01, Figure 6 C,D). However, the pre‐treatment of MK2206 significantly abated the Ori‐induced increase of 2‐NBDG uptake in insulin‐resistant PC12 cells (*P* < .01, Figure 6 E) and in insulin‐resistant N2a cells (*P* < .01, Figure 6 F). These results indicated that Ori improved IR via PTEN/p‐Akt/autophagy pathway.

### 3.6 Ori enhances synaptic formation via PTEN/Akt/autophagy pathway in insulin‐resistant cells

**Figure 7:**
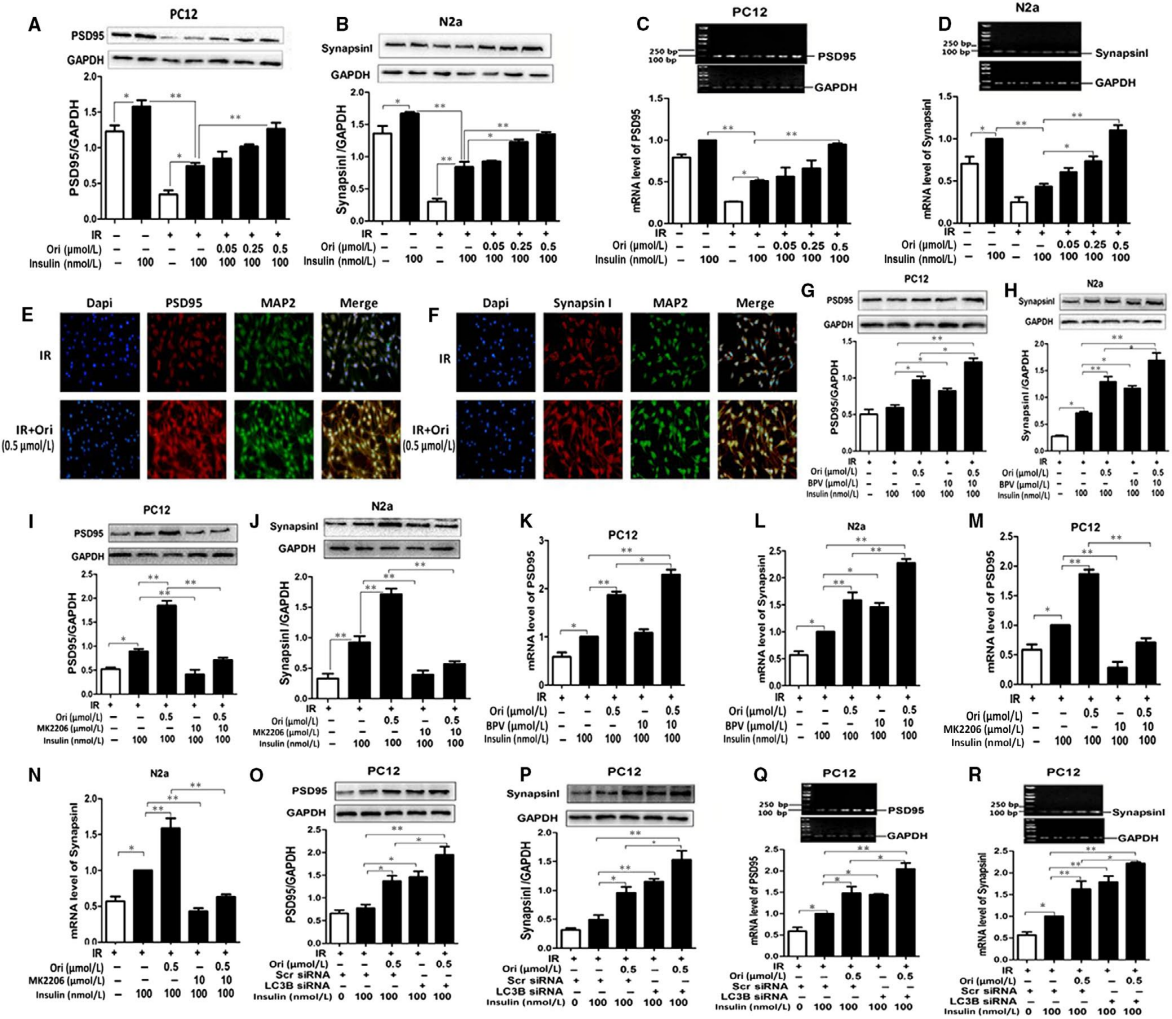
Ori enhances synaptic formation via PTEN/Akt/autophagy pathway in insulin‐resistant cells. A,B, IB analysis of lysates of PC12 (A) and N2a (B) cells treated with various concentration of Ori (0.05, 0.25, 0.5, 1.5 or 5 µmol/L) in the pre‐incubation of 3 µmol/L insulin together with 100 nmol/L insulin using anti‐PSD95/GAPDH (A) and antisynapsin I/GAPDH (B) antibodies and subsequent densitometry. C,D, RT‐PCR analysis for PSD95/synapsin I mRNA of PC12 (C) and N2a (D) cells treated with various concentration of Ori (0.05, 0.25, 0.5, 1.5 or 5 µmol/L) in the pre‐incubation of 3 µmol/L insulin together with 100 nmol/L insulin. E,F, Double immunofluorescence staining of PC12 cells treated with 0.5 µmol/L Ori in the pre‐incubation of 3 µmol/L insulin together with 100 nmol/L insulin using antibodies against PSD95 (E)/synapsin I (F) (red), MAP2 (green). G‐J, IB analysis of lysates of PC12 (G, I) and N2a (H, J) cells treated with 0.5 µmol/L Ori in the pre‐incubation of 3 µmol/L insulin together with 100 nmol/L insulin in the presence or absence of BPV or MK2206 using anti‐PSD95/GAPDH (G, I), antisynapsin I/GAPDH (H, J) antibodies and subsequent densitometry. K‐N, RT‐PCR analysis for PSD95/synapsin I mRNA of PC12 (K, M) and N2a (L, N) cells treated with 0.5 µmol/L Ori in the pre‐incubation of 3 µmol/L insulin together with 100 nmol/L insulin in the presence or absence of BPV or MK2206. O‐P, IB analysis of PC12 cells treated with 0.5 µmol/L Ori in the pre‐incubation of 3 µmol/L insulin together with 100 nmol/L insulin after LC3B siRNA transfection using anti‐PSD95/GAPDH (O), antisynapsin I/GAPDH (P) antibodies and subsequent densitometry. Q‐R, RT‐PCR analysis for PSD95/synapsin I mRNA of PC12 cells treated with 0.5 µmol/L Ori in the pre‐incubation of 3 µmol/L insulin together with 100 nmol/L insulin after LC3B siRNA transfection. Data are shown as mean ± SD. *P < .05, **P < .01. Scale bar, 25 μm. MRGD, merged image

IR would lead to impaired long‐term potentiation (LTP), decreased synaptic plasticity and resulting in impaired learning and memory.^47^, ^48^ Therefore, we tested whether Ori had the effect on IR‐mediated synaptic loss. By IB analysis, in PC12 cells, under normal condition, insulin stimulated an increase over the basal cellular level in PSD95 (*P* < .05, Figure 7 A). Under high concentration of insulin condition, insulin stimulated a slight but significant elevation of PSD95 expression compared to basal level (*P* < .05, Figure 7 A), while insulin failed to increase PSD95 expression compared to normal condition (*P* < .01, Figure 7 A), and treatment of Ori at 0.5 μmol/L caused notable increment in PSD95 (*P* < .01, Figure 7 A). In N2a cells, under normal condition, insulin stimulated an elevation over the basal cellular level in synapsin I (*P* < .05, Figure 7 B). Under high concentration of insulin condition, insulin stimulated a minor but notable increase of synapsin I expression compared to basal level (*P* < .05, Figure 7 B); however, insulin was unable to increase synapsin I expression compared to normal condition (*P* < .01, Figure 7 B), and addition of Ori at concentration of 0.25 and 0.5 μmol/L caused significant elevation of the expression of synapsin I (*P* < .05, *P* < .01, Figure 7 B). Then, we investigated the effects of Ori on the mRNA expression of the PSD95 and synapsin I. In PC12 cells, under normal condition, insulin stimulated a minor increase over the basal cellular level in PSD mRNA (*P* > .05, Figure 7 C). Under high concentration of insulin condition, insulin stimulated minor but notable elevation of PSD95 mRNA compared to basal level (*P* < .05, Figure 7 C), insulin was unable to increase in PSD95 mRNA compared to normal condition (*P* < .01, Figure 7 C), and treatment of Ori at 0.5 μmol/L markedly enhanced in PSD95 mRNA expression (*P* < .01, Figure 7 C). In N2a cells, under normal condition, insulin stimulated an obvious increase over the basal cellular level in synapsin I mRNA (*P* < .05, Figure 7 D). Under high concentration of insulin condition, insulin had few effects on synapsin I compared to basal level, while insulin failed to enhance synapsin I mRNA compared to normal condition (*P* < .01, Figure 7 D), and synapsin I mRNA expressions were significantly elevated induced by Ori at concentration of 0.25 and 0.5 μmol/L (*P* < .05, *P* < .01, Figure 7 D). IF staining showed that the reduction in PSD95 and synapsin I immunofluorescences was reversed by 0.5 μmol/L Ori treatment in insulin‐resistant PC12 cells (Figure 7 E,F). These results suggested that Ori had the ability to improve IR‐induced synaptic loss.

Study revealed that the inhibition of PTEN along with the activation of it downstream Akt phosphorylation restored synaptic protein synthesis and inactivation of autophagy resulted in the improvement of learning and memory performance.^24^, ^49^ Thus, we tested the effect of Ori on IR‐induced synaptic loss was associated with PTEN/Akt/autophagy signalling. First, we analysed the effect of Ori on synaptic formation through PTEN/Akt pathway. By IB analysis, high insulin‐induced, insulin‐resistant PC12 and N2a cells were pre‐treated with BPV or MK2206 for 2 hours followed by the addition of 0.5 μmol/L Ori for 36 hours. As expected, treatment of BPV caused further elevation of PSD95 expressions in Ori‐treated insulin‐resistant PC12 cells (*P* < .05, Figure 7 G), and BPV enhanced synapsin I expression in Ori‐treated insulin‐resistant N2a cells (*P* < .05, Figure 7 H). However, MK2206 significantly abated the Ori‐induced increase in PSD95 expression in insulin‐resistant PC12 cells (*P* < .01, Figure 7 I) and markedly diminished the increase in the expression of synapsin I in insulin‐resistant N2a cells exposed to Ori (*P* < .01, Figure 7 J). RT‐PCR analysis showed that BPV caused further elevation of PSD95 mRNA in Ori‐treated insulin‐resistant PC12 cells (*P* < .05, Figure 7 K) and amplified synapsin I mRNA expression in Ori‐treated insulin‐resistant N2a cells (*P* < .05, Figure 7 L). However, MK2206 significantly abated the Ori‐induced elevation of PSD95 mRNA in insulin‐resistant PC12 cells (*P* < .01, Figure 5 M), and Ori‐induced elevation of synapsin I mRNA was also diminished by MK2206 in insulin‐resistant N2a cells (*P* < .01, Figure 7 N). Then, we studied the effect of Ori on synaptic formation through autophagy in insulin‐resistant PC12 cells. IB analysis showed that knockdown of LC3B caused notable further increase in PSD95 and synapsin I in insulin‐resistant PC12 cells with treatment of Ori (*P* < .05, Figure 7 O,P). RT‐PCR analysis also showed that knockdown of LC3B amplified PSD95 and synapsin I mRNA expression in Ori‐treated insulin‐resistant PC12 cells (*P* < .05, Figure 7 Q,R). Thus, these results indicated that Ori was able to improve IR‐induced synaptic deficit via PTEN/Akt/autophagy signalling.

### 3.7 Effect of Ori on PTEN/AKT‐autophagy pathway in MHE rats

**Figure 8:**
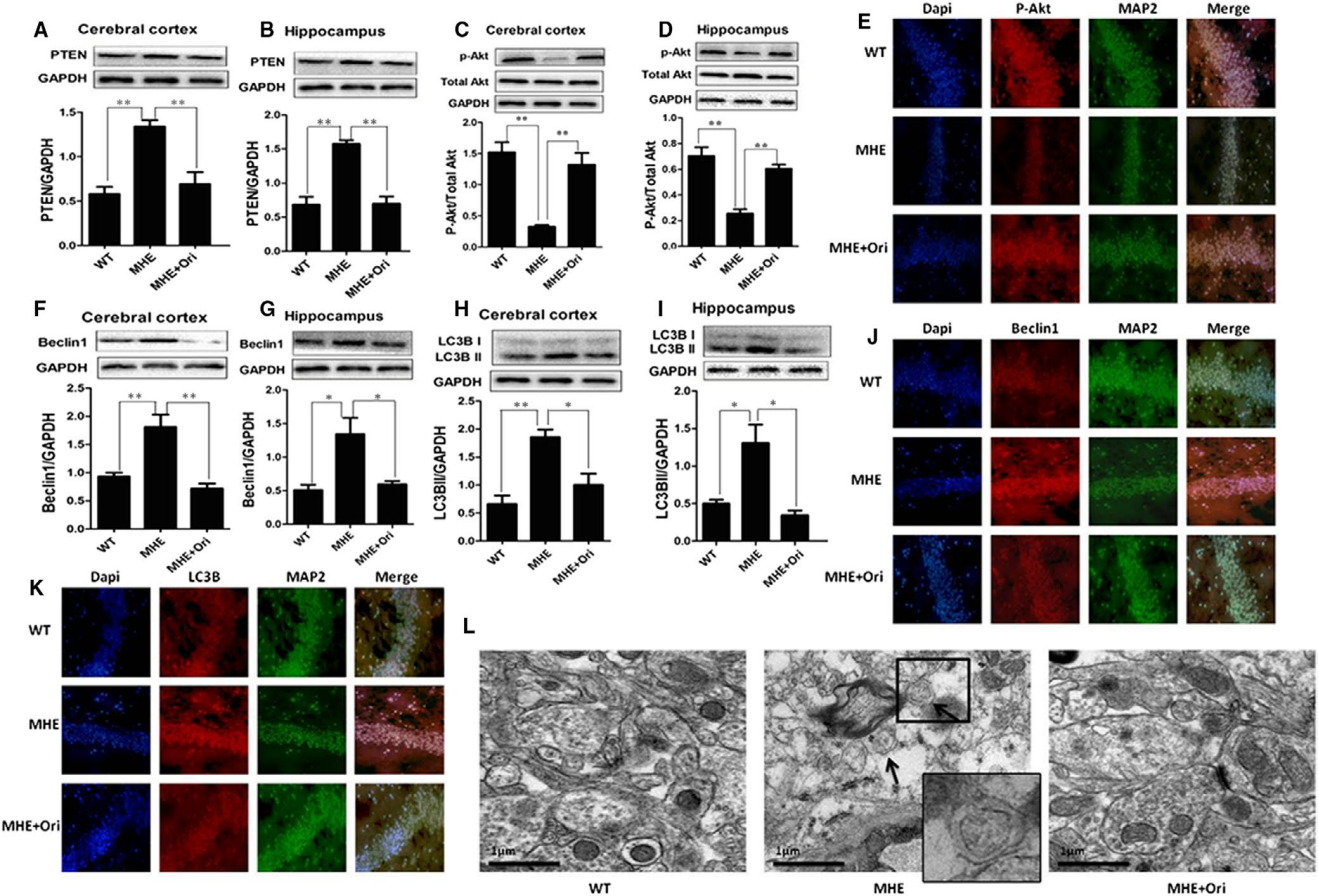
Effect of Ori on PTEN/AKT/autophagy pathway in MHE rats. A, IB analysis of cortical lysates from MHE rats treated with Ori using anti‐PTEN/GAPDH antibodies and subsequent densitometry. B, IB analysis of hippocampal lysates from MHE rats treated with Ori using anti‐PTEN/GAPDH antibodies and subsequent densitometry. C, IB analysis of cortical lysates from MHE rats treated with Ori using anti‐pAkt (Ser473)/total‐Akt antibodies and subsequent densitometry. D, IB analysis of hippocampal lysates from MHE rats treated with Ori using anti‐pAkt (Ser473)/total‐Akt antibodies and subsequent densitometry. E, Double immunofluorescence staining of the hippocampus from MHE rats treated with Ori using antibodies against pAkt (red), MAP2 (green). Scale bar, 25 μm. F, IB analysis of cortical lysates from MHE rats treated with Ori using anti‐Beclin1/GAPDH antibodies and subsequent densitometry. G, IB analysis of hippocampal lysates from MHE rats treated with Ori using anti‐Beclin1/GAPDH antibodies and subsequent densitometry. H, IB analysis of cortical lysates from MHE rats treated with Ori using anti‐LC3B/GAPDH antibodies and subsequent densitometry. I, IB analysis of hippocampal lysates from MHE rats treated with Ori using anti‐LC3B/GAPDH antibodies and subsequent densitometry. J, K, Double immunofluorescence staining of the hippocampus from MHE rats treated with Ori was using antibodies against Beclin1/LC3B (red), MAP2 (green). Scale bar, 25 μm. L, Assay for autophagosomes of hippocampal CA1 region from MHE rats treated with Ori using electron microscopy. A high magnification image of the indicated portion was shown at the right panel. The black arrows indicate typical autophagosomes. Data are shown as mean ± SD (n = 4). *P < .05, **P < .01. Scale bar, 1 μm

According to the above result, we examined whether Ori was influenced on the activation of PTEN/AKT signalling pathway in vivo. As indicated by IB, the expression of PTEN was significantly increased in the cerebral cortex and hippocampus of MHE rats (*P* < .01, Figure 8 A,B), whereas Ori administration to MHE rats significantly reduced the expression of PTEN in the cerebral cortex and hippocampus (*P* < .01, Figure 8 A,B). The phosphorylations of Akt were decreased in the cerebral cortex and hippocampus of MHE rats (*P* < .01, Figure 8 C,D), which were reversed by Ori administration (*P* < .01, Figure 8 C,D). Moreover, we tested the expression of p‐Akt by double immunofluorescence. IF staining showed the obvious decrease in p‐Akt immunofluorescence in the neurons in hippocampus of MHE rats, whereas Ori administration increased the p‐Akt immunofluorescence (Figure 8 E).

Then, we examined whether Ori had the effect on autophagy in vivo. By IB analysis, expressions of Beclin1 and LC3B were markedly increased in the cerebral cortex and hippocampus of MHE rats (*P* < .01, *P* < .05, Figure 8 F‐I), and Ori administration reversed expressions of two proteins (*P* < .01, *P* < .05, Figure 8 F‐I). By double IF staining, Beclin1 (Figure 8 J) and LC3B (Figure 8 K) immunofluorescences were increased in the neurons of hippocampus of MHE rats. Ori administration markedly decreased the Beclin1 and LC3B immunofluorescence. Then, we assessed autophagosomes formation by using electron microscopy. Electron microscopic examination of the hippocampus CA1 areas demonstrated notable increase in autophagosomes in MHE rats, supporting the biochemical alterations in key autophagy molecules (Figure 8 L). After administration with Ori, hippocampus of MHE rats exhibited the decrease in autophagosome (Figure 8 L). These data suggested that Ori improved PTEN/Akt/autophagy in MHE rats.

### 3.8 Ori prevents synaptic loss in MHE rats

**Figure 9:**
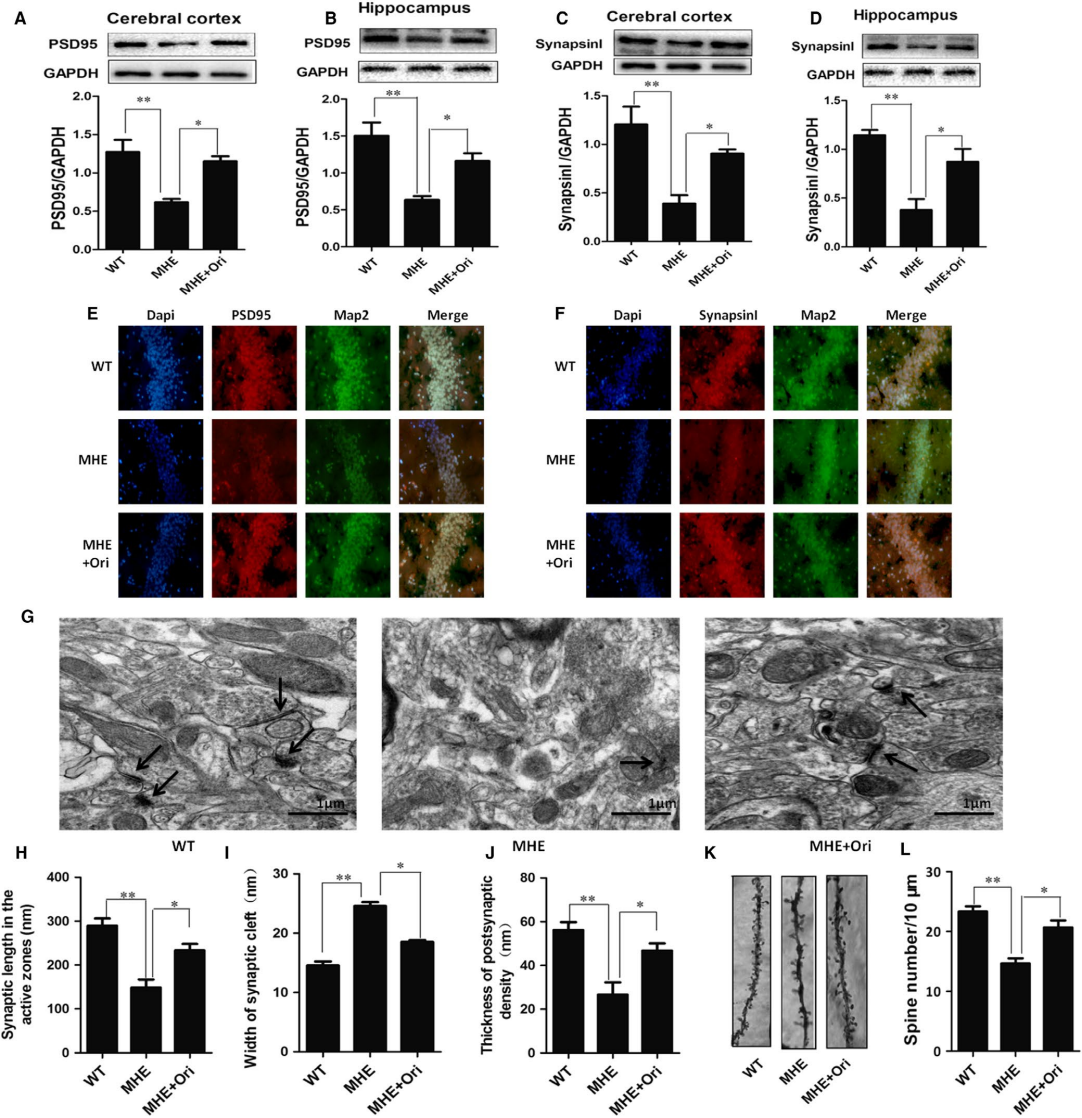
Ori prevents synaptic loss in MHE rats. A, IB analysis of cortical lysates from MHE rats treated with Ori using anti‐PSD95/GAPDH antibodies and subsequent densitometry. B, IB analysis of hippocampal lysates from MHE rats treated with Ori using anti‐PSD95/GAPDH antibodies and subsequent densitometry. C, IB analysis of cortical lysates from MHE rats treated with Ori using antisynapsin I/GAPDH antibodies and subsequent densitometry. D, IB analysis of hippocampal lysates from MHE rats treated with Ori using antisynapsin I/GAPDH antibodies and subsequent densitometry. E,F, Double immunofluorescence staining of the hippocampus from MHE rats treated with Ori using antibodies against PSD95 (E)/synapsin I (F) (red), MAP2 (green). G, The synaptic structures assay of hippocampal CA1 region of MHE rats treated with Ori using electron microscopy. Arrows indicate the synapses. Scale bar, 1 μm. H‐J, Quantitative analysis for ultrastructure synapses of MHE rats treated with Ori via synaptic length in the active zones (H), width of synaptic cleft (I) and thickness of post‐synaptic (J). K, Golgi staining of apical dendritic layer of the CA1 region in MHE rats treated with Ori. Scale bar, 5 mm. L, Quantitative analysis for the spine density of MHE rats treated with Ori. Data are shown as mean ± SD (n = 4), *P < .05, **P < .01. Scale bar, 25 μm. MRGD, merged image

It has been demonstrated that neurotoxicity and synaptic dysfunction were alleviated with the administration of Ori in Alzheimer's disease mice.^34^, ^50^ Synaptic loss is believed to be the basis of cognitive impairment in the MHE.^51^ We further confirmed the effect of Ori on the synaptic formation in vivo. By IB analysis, MHE rats showed a significant decrease in expressions of PSD95 and synapsin I in the cerebral cortex and hippocampus (*P* < .01, Figure 9 A‐D), and Ori treatment reversed the expressions of the two synaptic markers in the cerebral cortex and hippocampus (*P* < .05, Figure 9 A‐D). As indicated by the double IF analysis which are labelled of PSD95/synapsin I and Map2 in Figure 9 E,F, we confirmed that immunofluorescences of both PSD95 and synapsin I were significantly reduced in the hippocampus of MHE rats, while Ori administration increased immunofluorescences of the two proteins.

Next, we quantified the density and ultrastructure of synapses in the CA1 area of all groups’ brains by electron microscopy. We found that synaptic density was significantly reduced in MHE rats, while Ori administration increased the synaptic density (Figure 9 G). And ultrastructure of synapses was impaired in MHE rats, shorten synaptic length in the active zones (*P* < .01, Figure 9 H), increased width of synaptic cleft (*P* < .01, Figure 9 I) and reduced thickness of post‐synaptic (*P* < .01, Figure 9 J). Ori treatment significantly improved ultrastructure of synapses (*P* < .05, Figure 9 H‐J). As each dendritic spine can form more than one synapse, so we assessed the density of dendritic spines along individual dendrites of pyramidal neurons by Golgi staining. The densities of dendritic spines were markedly decreased in MHE rats (*P* < .01, Figure 9 K,L), and this deficit was obviously rescued by administration with Ori (*P* < .05, Figure 9 K,L). These data suggested that Ori improved synaptogenesis in MHE rats.

## 4 DISCUSSION

Emerging evidence demonstrated that IR could cause learning and memory impairment.^52^, ^53^ And studies found that antidiabetic drug could improve metabolic parameters and cognition in insulin‐resistant rats.^54^, ^55^ In this study, we found Ori is able to increase insulin sensitivity in insulin‐resistant cells and MHE rats. IR stimulates cognitive decreased by at least 2‐fold,^56^ and also IR would disrupt the function of the brain vasculature.^57^, ^58^ Impaired glucose tolerance and hyperinsulinaemia are regular features in liver cirrhosis.^59^, ^60^, ^61^ Our previous study had proved that brain IR exists in MHE, and IR could induce cognitive decline in MHE.^14^ In the present study, we evaluated cognitive function using MWM and Y‐M test. The MWM and Y‐M protocol is a spatial discriminative cognitive model, which is related to the function and structure of hippocampus. The memory and learning ability of MHE rats reflected by MWM and Y‐M performances were impaired, and rescued by Ori treatment. The results from this study, we found Ori could improve cognitive impairment and insulin sensitivity in MHE rats. Therefore, our study showed the underlying mechanism that Ori rescues IR‐mediated cognitive decline in MHE.

Evidence indicates that increased levels of the PTEN have the negative impact on insulin/Akt signalling 43 and negatively influence insulin sensitivity.^44^ As tissue‐specific deletion of PTEN in mice models prevented the development of IR and diabetes induced by high‐fat diet.^62^ The PTEN pathway is engaged in the regulation of insulin signalling, and PTEN in insulin resistance induced by high glucose concentrations in podocytes.^63^ Here, we found that the expressions of PTEN enhanced and pAkt decreased in insulin‐resistant cells, and Ori could reverse the elevated PTEN and stimulate the expression of pAkt. And PTEN inhibitor further stimulated the glucose uptake in Ori‐treated insulin‐resistant cells, but Akt inhibitor abated the effect of Ori‐induced increase of glucose uptake in insulin‐resistant cells. We also found some terpenoids such as ginsenosides have a stimulatory effect on insulin/Akt signalling in cells.^64^ In this regard, IR induced the impairment of PTEN/Akt pathway and reversed by Ori. Furthermore, studies found that dysfunction of Akt pathway participates in neurodevelopmental diseases with distinct clinical phenotype.^65^, ^66^ PTEN plays a negative role in PI3K/Akt signalling pathway, which is responsible for cell‐survival signalling pathway in neurons, so PTEN is an important factor of study for cognitive. In MHE rats’ hippocampal and cerebral cortex, the PTEN proteins elevated and pAkt proteins decreased, Ori decreased the expressions of PTEN and improved pAkt activity in MHE. Given the key roles that PTEN/Akt signalling has in memory, we propose that Ori may protect cognitive decline in MHE rats through IR‐mediated PTEN/Akt pathway.

Autophagy impairment would destroy hepatic glucose homeostasis and insulin sensitivity in diabetic. According to the underlying reported role in islet function and survival,^67^, ^68^ the results suggested that autophagy may be linked to the mechanism of two main pathological targets of IR: dysfunction in insulin secretion and insulin sensitivity. This statements in similar to our findings in the present study, as shown in our results, in insulin‐resistant cells, the expressions of autophagy proteins increased markedly. However, the high insulin‐induced autophagy overexpression in insulin‐resistant cells was blocked by the Ori. The excessive or abnormal autophagy can exert detrimental effects in the CNS that are thought to contribute to the pathogenesis of several neurodegeneration diseases.^69^, ^70^, ^71^ Thus, IR induces the excessive autophagy may lead to the learning impairment in MHE. The TEM results of the current study showed that MHE rats exhibited pathologic abnormalities of increased autophagic vacuole in the neurons of the hippocampal CA1 region, which is reduced by Ori‐treated. And autophagy was associated with two key markers: an elevated LC3‐II/LC3‐I ratio and enhanced Beclin1 expression in MHE rats’ cerebral cortex and hippocampal, which decreased in Ori‐treated MHE rats. Our studies had provided evidence that Ori could suppress autophagy which induced by IR in MHE rats.

PTEN has also been shown to control autophagy in mammalian cells against the inhibitory effect of the PI3K/Akt pathway mediated by autophagic.^72^ In this regard, PTEN has been widely recognized as a positive regulator of autophagy through inhibition of Akt.^73^ Ori significantly decreased LC3B and Beclin1 in insulin‐resistant cells, suggesting that Ori has an ability to improve autophagy. This effect was stimulated by PTEN inhibitor and abated by Akt inhibitor, indicating that the effect of Ori on the improvement of autophagy is PTEN/Akt dependent. And administration of Ori activated PTEN/Akt signalling and inactivated autophagy in the brain of MHE rats. Therefore, the current study was designed and performed with the hypothesis that Ori could prevent IR‐induced cognitive decline by mediating the PTENAkt‐autophagy pathway in MHE.

Synaptic dysfunction is a major pathophysiological hallmark in MHE and other neurodegenerative diseases. Ori has been reported that it could attenuate synaptic loss in a mouse model of Alzheimer's disease.^74^ In addition, Ori has been reported to have several neuroregulatory effects through modulation of multiple functions of nerve cells, which have been implicated as a potential agent in many neurological disorders.^75^ We also show that Ori promotes synaptogenesis. Conceivably, our in vitro results indicate that Ori may provide protective effects on synapses in insulin‐resistant cells. In vivo results, we observed a decrease in dendritic spine density in the hippocampus of MHE rats and that Ori elevated the spine density in apical dendrites of CA1 neurons of hippocampus. So the data indicated that Ori mitigated synaptic dysfunction in MHE rats. In line with this result, as IR is related to synaptic loss and cognitive impairment, our results showed that the attenuation of peripheral IR condition though Ori could ameliorate the cognitive dysfunction in MHE rats. And Ori enhanced synaptogenesis of neurons after high concentration of insulin injury in vitro. In accord with this observation, results suggest that Ori enhances insulin sensitivity to prevent the synaptic dysfunction and cognitive deficits in IR‐induced MHE.

It is revealed that PTEN exists an important role in the control of synaptic plasticity and cell survival under the pathological conditions 76, 77 and in addition activating PTEN related to synaptic dysfunction in hippocampal neurons.^78^ In insulin‐resistant neuron cells, PTEN inhibitor further enhanced the expression of synaptic proteins in Ori‐treated insulin‐resistant cells, and Akt inhibitor abated the Ori‐induced elevation of the expressions of synaptic. So Ori enhances synaptic formation via PTEN/Akt pathway in insulin‐resistant cells. Some studies found activation of autophagy would decline the major proteins involved in synaptic plasticity.^79^ In our study, we showed that the LC3B knockout Ori‐treated insulin‐resistant cells resulted in a further increasing of synaptic protein expression levels, indicating that decreased autophagy could reactivate synaptic formation in IR state of cells. Nevertheless, the in vitro results suggested that Ori may be used to protect these synaptic losses mediated by IR through preventing autophagy. Thus, we suppose that maybe Ori rescue synaptic express through improving IR‐mediated PTEN/Akt‐autophagy pathway, and improve the cognitive decline in MHE.

In summary, this study demonstrated that administration of Ori could reverse the IR‐induced cognitive decline that was associated with an activity of PTEN/pAkt/autophagy signalling in the MHE rats.

### 4.1 Limitations

Our study might identify Ori as a novel potential cognitive protective strategy for the treatment of some neurodegenerative diseases, but there are still need lots of data and experiments to support it. Ori has become one of the most popular herbs used clinically for the treatment of inflammatory diseases, antibacterial and antitumour. However, Ori has not been used in clinic for treatment of neurodegenerative diseases. So, in our later work, we will concentrate on doing more research to get enough data and making it used in the clinic trail to verify it.

## CONFLICT OF INTEREST

The authors have declared no conflict of interest.

## AUTHOR CONTRIBUTION

Ding S and Zhuge Q supervised the entire project and designed the research. WEN F conceived and designed the experiments, performed the research interpreted, and analysed the data, and wrote the paper. Zhuge W and Wang J conceived and designed the experiments, interpreted and analysed the data, and supervised all the experimental procedure. Lu X and You R performed the research and analysed the data. Liu L analysed the data and critically revised the manuscript. All authors read and approved the final manuscript.

## Data Availability

The data that support the findings of this study are available from the corresponding author upon reasonable request.
